# Nature and reporting characteristics of UK health technology assessment systematic reviews

**DOI:** 10.1186/s12874-018-0498-6

**Published:** 2018-05-08

**Authors:** Christopher Carroll, Eva Kaltenthaler

**Affiliations:** School of Health and Related Research (ScHARR), University of Sheffield, Regent Court, Regent, Street, Sheffield, S1 4DA UK

**Keywords:** Systematic review, Health technology assessment, HTA, Cochrane collaboration, Reporting, PRISMA

## Abstract

**Background:**

A recent study by Page et al. (PLoS Med. 2016;13(5):e1002028) claimed that increasing numbers of reviews are being published and many are poorly-conducted and reported. The aim of the present study was to assess how well reporting standards of systematic reviews produced in a Health Technology Assessment (HTA) context compare with reporting in Cochrane and other ‘non-Cochrane’ systematic reviews from the same years (2004 and 2014), as reported by Page et al. (PLoS Med. 2016;13(5):e1002028).

**Methods:**

All relevant UK HTA programme systematic reviews published in 2004 and 2014 were identified. After piloting of the form, two reviewers each extracted relevant data on conduct and reporting from these reviews. These data were compared with data for Cochrane and “non-Cochrane” systematic reviews, as published by Page et al. (PLoS Med. 2016;13(5):e1002028). All data were tabulated and summarized.

**Results:**

There were 30 UK HTA programme systematic reviews and 300 other systematic reviews, including Cochrane reviews (*n* = 45). The percentage of HTA reviews with required elements of conduct and reporting was frequently very similar to Cochrane and much higher than all other systematic reviews, e.g. availability of protocols (90, 98 and 16% respectively); the specification of study design criteria (100, 100, 79%); the reporting of outcomes (100, 100, 78%), quality assessment (100, 100, 70%); the searching of trial registries for unpublished data (70, 62, 19%); reporting of reasons for excluding studies (91, 91 and 70%) and reporting of authors’ conflicts of interests (100, 100, 87%). HTA reviews only compared less favourably with Cochrane and other reviews in assessments of publication bias.

**Conclusions:**

UK HTA systematic reviews are often produced within a specific policy-making context. This context has implications for timelines, tools and resources. However, UK HTA systematic reviews still tend to present standards of conduct and reporting equivalent to “gold standard” Cochrane reviews and superior to systematic reviews more generally.

**Electronic supplementary material:**

The online version of this article (10.1186/s12874-018-0498-6) contains supplementary material, which is available to authorized users.

## Background

Systematic reviews are seen as having an important role in decision-making processes, especially in medicine and health, and the numbers of published reviews are increasing [1]. Under such circumstances, the issue of the quality of these reviews is also becoming increasingly important. A recent analysis of the epidemiology and reporting characteristics of systematic reviews by Page et al. [1] found that the quality of the conduct and reporting of many reviews was often poor. This finding was based on an assessment of the reporting of 300 systematic reviews published in a single month in 2014 and, in part, by comparison with data on the reporting of 300 reviews published in the equivalent month in 2004 [2]. The research concluded that reporting had improved since 2004, in large part due to developments such as the publication of the PRISMA (Preferred Reporting Items for Systematic Reviews and Meta-analyses) statement [3] as the relevant standard for the reporting of systematic reviews. However, it noted that poor conduct and reporting was still frequent (indeed, it was considered to be ‘suboptimal’ for many characteristics) and strategies are needed to improve this if systematic reviews are to offer genuine rather than just hypothetical value to ‘patients, health care practitioners and policy-makers’ [1]. This is important because the results of poorly-conducted, reported or flawed systematic reviews include ‘misleading conclusions’, which can have major implications for decision-making [1]. Consequently, Page et al. made a series of highly appropriate and manageable recommendations to improve the conduct and reporting of systematic reviews generally, and thus to re-establish the method as trustworthy and robust as far as possible [1]. These included using certain ‘writing tools’ to enforce reporting guidelines; editorial application of guidelines; involvement of relevant stakeholders from beyond the review team; and assessing the reporting of bias within the systematic review itself [1].

The work also concluded that Cochrane reviews were ‘superior’ to ‘non-Cochrane’ reviews in terms of the ‘completeness’ of their reporting. This difference in relative quality has been found in other work too, though with a rather more critical assessment of the Cochrane reviews themselves [4]. However, it is questionable whether ‘non-Cochrane’ reviews represent a homogenous group: they are published in many different journals and formats. Some ‘non-Cochrane’ systematic reviews are commissioned by policy-makers, or programmes related to policy-makers, and as such already involve many safeguards against the reporting of potentially ‘misleading conclusions’. Health Technology Assessment (HTA) represents a specific field in which the systematic review of clinical effectiveness evidence plays a fundamental role in healthcare decision-making [5, 6]. These systematic reviews therefore represent a distinct type of ‘non-Cochrane’ review. One such group of systematic reviews are those conducted for the UK National Institute of Health Research, (NIHR) HTA programme; the full versions of these systematic reviews are published in that programme’s own journal series, Health Technology Assessment (Winchester) [7]. This series of ‘non-Cochrane’ systematic reviews represents an interesting and informative comparison with Cochrane reviews given that, like Cochrane reviews, these reviews have clear reporting standards and are not constrained by word limits or the absence of online appendices unlike many other ‘non-Cochrane’ reviews published in more conventional peer-reviewed journals, an issue acknowledged by Page et al. [1]. In fact, Page et al. do acknowledge that the apparent bias in quality of reporting in favour of Cochrane reviews might not always be bias in the conduct or reporting of the reviews at all, but rather might be due to the restriction of word limits [1].

The aim of this research therefore is to compare the reporting of systematic reviews published in the UK Health Technology Assessment journal series with the reporting of systematic reviews generally, and Cochrane reviews specifically, from the years 2004 and 2014, as reported by Page et al. [1]. A brief assessment will be made of developments between 2004 and 2014 for UK HTA systematic reviews, but the key comparison is across the UK HTA, Cochrane and ‘non-Cochrane’ reviews from 2014 because this offers the best evidence on current practice and standards. This comparison is important because it seeks to underline not only how so-called ‘non-Cochrane reviews’ are not all the same, but that the context in which systematic reviews are produced is a key driver in standards of conduct and reporting. Further, some of the recommendations for improved practice made by Page et al. are arguably already being met by the staff and editorial processes involved in the production of the Health Technology Assessment journal systematic reviews, and so a comparison with these systematic reviews might potentially offer further support for the authors’ proposals.

## Methods

The data from the Cochrane and ‘non-Cochrane’ systematic review samples from a single month in each of 2004 and 2014 were published in the paper by Page et al. [1]. In order to generate the data for the proposed comparison between UK HTA systematic reviews and the systematic reviews reported in Page et al., [1], a simple search was performed in August 2016 to identify the systematic reviews published in the Health Technology Assessment monograph series from 2004 and 2014. This involved a structured search of MEDLINE combining title or abstract terms for ‘systematic review’ with the Health Technology Assessment journal identifier (‘health technology assessment winchester england.jn’), and the application of date limits (2004 and 2014). The current paper has included Health Technology Assessment systematic reviews from the whole of the designated years (rather than just one month) in order to generate a sufficiently sizeable sample from this single journal for comparison with the data published by Page et al. [1]. All of the systematic reviews were published in English. The searching and screening process was conducted by one reviewer (CC) and checked by a second (EK).

Data extraction used a version of the form published by Page et al. [1] with some modifications, e.g. the addition of a ‘Not Applicable’ option. The form was piloted by the two reviewers (CC, EK) on four Health Technology Assessment journal systematic reviews to enhance consistency and accuracy of extraction. Each reviewer then extracted half of the Health Technology Assessment systematic reviews. The data extracted included type of review, number of included studies, availability of protocols, details of search strategies, inclusion criteria, critical appraisal tools, synthesis methods etc.. Data were summarized as frequencies and percentages for categorical items and as median and range for continuous items. Results from the Health Technology Assessment systematic reviews from 2004 and 2014 were tabulated alongside the previously published results from the Cochrane reviews and ‘non-Cochrane’ systematic reviews from the same years from the paper by Page et al. [1] These data were then compared and discussed.

## Results

The MEDLINE search for Health Technology Assessment journal systematic reviews found 462 unique citations. Twenty-two were Health Technology Assessment publications with a systematic review of the clinical evidence from 2004 and 30 were equivalent publications from 2014. The total number of Health Technology Assessment monographs to be extracted therefore was 52, but one monograph contained two relevant reviews [8], so the total number of reviews extracted was 53. A list of the included reports is available in Additional file 1. The number of 30 UK HTA systematic reviews for 2014 compares reasonably with the sample of 45 Cochrane reviews from 2014. The Page et al. study also reported on 255 non-Cochrane reviews from 2014 and 300 systematic reviews published in 2004 [1]. This latter sample, and the ‘non-Cochrane’ reviews sample from 2014, might have included some of the Health Technology Assessment journal systematic reviews, but the detailed data are not available to confirm this or to establish what proportion of the Health Technology Assessment journal systematic reviews were included.

### Disease areas

Fifty of the 53 systematic reviews covered seventeen different disease areas (the remaining three publications were reviews of methods [9–11]). The disease areas covered by the Health Technology Assessment reviews were principally neoplasms (16%), diseases of the circulatory systems (16%), and endocrine, nutritional or metabolic diseases (14%). The proportions of Health Technology Assessment systematic reviews covering certain disease areas were mostly similar to those found for systematic reviews generally in 2014. A full list is provided in Table 1.

**Table 1:** Disease areas covered by included systematic reviews (the HTA data were collected for this study; the other data are from Page et al. [1])

Disease area	HTAs 2004 & 2014	Systematic reviews 2004 [1]^a^	Systematic reviews 2014 [1]^a^
Neoplasms (i.e. cancers, carcinomas, tumours)	8 (16%)	22 (7%)	49 (16%)
Diseases of the circulatory system	8 (16%)	33 (11%)	34 (11%)
Endocrine, nutritional or metabolic disease	7 (14%)	NR	23 (8%)
Mental and Behavioural	5 (10%)	40 (13%)	22 (7%)
Infections and parasitic diseases	3 (6%)	22 (7%)	41 (14%)
Diseases of the nervous system	3 (6%)	NR	NR
Musculoskeletal	3 (6%)	NR	20 (7%)
Diseases of the eye and adnexa	2 (4%)	NR	NR
External causes of mortality and morbidity	2 (4%)	NR	NR
Genito-urinary	2 (4%)	NR	NR
Diseases of the digestive system	2 (4%)	20 (7%)	25 (8%)
Ear and mastoid process	1 (2%)	NR	NR
Pregnancy and birth	1 (2%)	NR	NR
Blood	1 (2%)	NR	NR
Skin and subcutaneous tissue	1 (2%)	NR	NR
Congenital malformations	1 (2%)	NR	NR
Total	50 (100%)	137^b^	214^b^

*HTA* Health Technology Assessment; ^a^As reported by Page et al. [1] ^b^Page et al. did not report values less than 7% so not all 300 systematic reviews are included; NR: Not reported

### Topics and administrative information

In terms of the basic distribution of the reviews, Cochrane reviews in 2014 exclusively covered therapeutic topics, while just over half of Health Technology Assessment and other ‘non-Cochrane’ reviews did so (53 and 55% respectively): see Table 2. This was less than 2004, when the respective proportions were 70 and 71%. More than half of the Cochrane reviews in 2014 evaluated pharmacological interventions, compared with 20% of HTA systematic reviews from the same year. The type of reviews conducted for the UK HTA programme was also different in 2004 and 2014: by 2014 Health Technology Assessment reviews in particular were much broader than Cochrane or other non-Cochrane reviews in their remit, covering therapies, diagnostics, prognostics and prevention, epidemiology and methodology. One quarter (25%) of other non-Cochrane reviews focused on epidemiology.

**Table 2:** Basic distribution characteristics and administrative information of included systematic reviews (the HTA data were collected for this study; all other data are from Page et al. [1], i.e. the data collected for 300 systematic reviews for a single month in 2004 and 2014)

Characteristic	2004 *n* = 300 [1]	HTA 2004 *n* = 23	HTA 2014 *n* = 30	Cochrane 2014 *n* = 45 [1]	2014 *n* = 300 [1]
Number of authors: Median and range ^a^	4 (1- > 7)	4 (2–8) ^b^	4 (1–9) ^b^	4 (3–6)	5 (4–6)
Update of previous SR	- (18%)	1 (4)	2 (7)	25 (56%)	31 (10%)
Type of SR					
Therapeutic	213 (71%)	16 (70%)	16 (53%)	45 (100%)	164 (55%)
Diagnostics / prognostics	23 (8%)	5 (22%)	10 (34%)	0	33 (11%)
Epidemiology	38 (13%)	2 (8%)	1 (3%)	0	74 (25%)
Other (incl. methodological)	46 (15%)	0	3 (10%)	0	29 (10%)
Type of intervention					
Pharmacological	- (47%)	13 (57%)	6 (20%)	23 (51%)	76/164 (46%)
Non-pharmacological	- (38%)	3 (13%)	13 (43%)	17 (38%)	75/164 (46%)
Both	NR	1 (4%)	2 (7%)	5 (11%)	13/164 (8%)
Not applicable (e.g. diagnostic test)	NR	6 (26%)	9 (30%)	0	0
Reporting guideline cited	NR	5/23 (24%)	19/30 (63%)^c^	1 (2%)	87 (29%)
Cochrane methods used	NR	1/23 (4%)	4/30 (13%)	45 (100%)	138 (46%)
Number of included studies	16	(1–170) [1–84]	0–200	9 (4–17)	15 (8–25)
Number of included participants reported	1112	2 only (2905–3909)	5 only (201–34,082)	1113 (421–2751)	2072 (672–8033)
Empty review (no eligible studies)	NR	0	2 (7%)	3 (7%)	4 (1%)
Meta-analysis performed	- (54%)	11 (48%)	15 (50%)	32 (71%)	189 (63%)
Number of studies included in largest meta-analysis	NR	4–17 (*n* = 11)	2–35 (*n* = 15)	6 (3–11)	9 (6–17)
Harms considered (excluding empty / diagnostic reviews; treatment reviews only)	- (75%)	15/16 (94%)	10/14 (71%)	41 (91%)	113/164 (69%)
Economics (i.e. costs) considered	- (24%)	0^d^	0^d^	7 (16%)	23/172 (13%)
SR or Meta-analysis mentioned in title / abstract	- (50%)	23 (100%)	30 (100%)	15 (33%)	254 (85%)
Review registered	NR	0/23 (0%)	23/30 (77%)	0 (0%)	12 (4%)
Protocol available	NR	4/23 (17%)	27/30 (90%)	44 (98%)	49 (16%)
Protocol mentioned but not available	NR	7/23 (30%)	1/30 (3%)	NR	NR
Conflicts of Interests reported					
Review authors	NR	23 (100%)	30/30 (100%)	45 (100%)	260 (87%)
Included studies’ authors	NR	6/23 (26%)	12/28 (43%)	13/42 (31%)	21/296 (7%)
Source of funding					
Not for profit	- (48%)	23 (100%)	30/30 (100%)	38 (84%)	142 (47%)
For profit	- (2%)	0	0	0 (0%)	8 (3%)
Mixed	- (6%)	0	0	0 (0%)	2 (1%)
No funding	- (1%)	0	0	5 (11%)	39 (13%)
Not reported	- (41%)	0	0	2 (4%)	109 (36%)

*HTA* Health Technology Assessment; ^a^Interquartile range for non-Health Technology Assessment data; ^b^Including information specialists, who are sometimes not listed as an author, but appear in the Acknowledgements; ^c^Some cite a combination of guidelines, e.g. PRISMA and York Centre for Reviews and Dissemination (CRD) guidance; ^d^Each clinical systematic review was accompanied by a separate cost-effectiveness systematic review and economic model; *NR* Nor reported

There are several similarities in the nature and reporting across the various systematic reviews. However, there are some notable differences too, as detailed in Table 2. These include the much larger range of included studies, with higher maximum numbers of included studies in the reviews and associated meta-analyses of the Health Technology Assessment reviews; the failure of Health Technology Assessment reviews to report the total number of participants involved in the studies included in a review (these reviews tend to report the number of participants across included studies as a range rather than a single total); the registering and availability of systematic review protocols; and a statement specifying that named guidelines had been followed in the conduct of the review. Reporting regarding protocols (an increase from 17 to 90%) and guidelines (an increase from 24 to 63%) differed markedly in the Health Technology Assessment sample between 2004 and 2014. However, it should also be noted that the reporting of the Health Technology Assessment systematic reviews compares favourably with the Cochrane reviews in terms of this administrative information– as well as information on conflicts of interest and funding - and is consistently more complete than the reporting of other so-called ‘non-Cochrane’ reviews. For example, for HTA, Cochrane and non-Cochrane reviews respectively, the availability of protocols was 90, 98 and 16%, and the reporting of authors’ conflicts of interests was 100, 100 and 87%.

Perhaps surprisingly there were relatively similar numbers of ‘empty’ systematic reviews (reviews without any included studies) across the different types of reviews: for reviews from 2014, there were three empty Cochrane reviews (7%); two empty Health Technology Assessments (7%), and four empty non-Cochrane / non-HTA reviews (1%). However, these figures are much lower than is known for Cochrane reviews generally [12]. Health Technology Assessment reviews are commissioned and conducted within a particular evidence-based and policy-making context, which permits the consideration of study designs other than RCTs or quasi-RCTs [12], so the presence of two ‘empty reviews’ in the 2014 sample is therefore unexpected. It is clear, however, that every effort was made in these reviews to find useable evidence from any comparative study design, but the only available data were from trials with completely different groups in each arm [13] or the complete absence of any relevant comparative studies at all, of any design [14].

### Inclusion criteria and search strategies

The 2014 HTA reviews were also consistently better at reporting on these criteria than other non-Cochrane reviews (see Table 3). Cochrane reviews were the best reported in terms of explicitly reporting on the inclusion of published and unpublished studies, but less explicit than HTA reviews in terms of reporting language criteria. Given the differing contexts in which the various reviews were produced, and their differing scopes, Cochrane reviews unsurprisingly included a much more restricted evidence base (89% of reviews only included randomized controlled trials [RCTs] or ‘quasi-RCTs’), while the non-Cochrane reviews included a much broader evidence base of study designs in order to answer the questions addressed by the reviews (including cohort studies for diagnostic reviews, for example). More HTA reviews in 2014 than 2004 explicitly reported whether the review included both published and unpublished studies, and whether it included studies in English only or other languages too.

**Table 3:** Eligibility criteria and searches (the HTA data were collected for this study; all other data are from Page et al. [1], i.e. the data collected for 300 systematic reviews for a single month in 2004 and 2014)

Characteristic	2004 *n* = 300 [1]	HTA 2004 *n* = 23	HTA 2014 *n* = 30	Cochrane 2014 *n* = 45 [1]	2014 *n* = 300 [1]
Type of included literature					
Published and unpublished^a^	- (41%)	9/23 (39%)	19 (65%)	41 (91%)	116 (39%)
Published only	- (23%)	0	1 (5%)	2 (4%)	80 (27%)
Not reported	- (36%)	14/23 (61%)	10 (33%)	2 (4%)	103 (34%)
All languages	- (37%)	11 (48%)	18 (60%)	37 (82%)	129 (43%)
English only	- (16%)	9 (39%)	11 (37%)	1 (2%)	92 (31%)
Not reported	- (45%)	3 (13%)	1 (3%)	6 (13%)	48 (16%)
Study design criteria specified^b^	- (72%)	21/23 (93%)	30/30 (100%)	45 (100%)	237 (79%)
RCTs	NR	15 (65%)	25 (83%)	44 (98%)	158 (53%)
Quasi-RCTs	NR	3 (13%)	11 (37%)	14 (31%)	33 (11%)
Controlled	NR	3 (13%)	12 (40%)	4 (9%)	30 (10%)
Cohort	NR	1 (4%)	12 (40%)	0 (0%)	76 (25%)
Case-control	NR	0 (0%)	4 (13%)	0 (0%)	49 (16%)
Other	NR	5 (22%)	4 (13%)	1 (2%)	56 (19%)
Unclear	NR	2 (9%)	2 (6%)	0 (0%)	36 (12%)
Only RCTs / Quasi-RCTs	NR	9 (39%)	8 (27%)	40 (89%)	107 (36%)
Number of databases median (range)	3	10 (4–15)	9 (2–13)	5 (4–6)	4 (3–5)
Start / end dates of all databases given	- (69%)	20/23 (87%)	28 (93%)	41 (91%)	196 (65%)
Only some start / end dates given	- (16%)	1/23 (4%)	2 (7%)	4 (9%)	88 (29%)
Full search given of ≥ 1 database	- (42%)	21/23 (91%)	30 (100%)	44 (98%)	134 (45%)
Other sources searched					
One or more trial registries^a^	NR	12 (52%)	21 (70%)	28 (62%)	58 (19%)
Number of other source types searched	NR	3 (1–6)	3 (1–6)	2 (1–3)	1 (1–2)
Grey literature	NR	8 (35%)	15 (50%)	9 (20%)	21 (7%)
Reference lists	NR	21 (91%)	26 (87%)	38 (84%)	243 (81%)
Conference abstracts	NR	7 (30%)	10 (33%)	11 (24%)	47 (16%)
Experts	NR	7 (30%)	16 (53%)	23 (51%)	54 (18%)
Handsearching particular journals	NR	5 (22%)	3 (10%)	6 (13%)	25 (8%)
Manufacturers	NR	14 (61%)	4 (13%)	8 (18%)	11 (4%)
Regulators	NR	3 (13%)	0 (0%)	0 (0%)	2 (1%)
Citation tracking	NR	2 (9%)	2 (7%)	8 (18%)	35 (12%)

*HTA* Health Technology Assessment; ^a^Note: there are some inconsistencies in the data because a search for ‘unpublished’ data might not be noted explicitly in the Methods of a Health Technology Assessment systematic review, but trial registers and grey literature are searched and often included and have much higher percentages ^b^Some of the figures do not add-up to 100% because, for example in study designs, a systematic review might include more than one design; *NR* Not reported

The reporting of the database searches represented one area where there was little difference between 2004 and 2014 in the HTA reviews: the reporting standards were extremely high in both years. In fact, the HTA reviews were much better reported than other non-Cochrane reviews and slightly superior also to Cochrane reviews in this sample, for example, in interrogating one or more trial registries.

### Screening, extraction and risk of bias assessment

In terms of the conduct and reporting of the study selection, data extraction and critical appraisal processes, the HTA reviews had some small differences between 2004 and 2014, and were consistently better-reported than the other non-Cochrane reviews (see Table 4). Respectively for data extraction and quality assessment, either two reviewers were reported to have completed the processes, or one reviewer conducted the process, with all data being checked by a second reviewer, in 93 and 88% of 2014 Cochrane reviews, compared with 86 and 79% of HTA reviews, and 65 and 52% of other ‘non-Cochrane’ reviews. The Cochrane reviews more frequently conducted these processes using two reviewers working independently (best practice), while standard practice in HTA reviews was generally to have the extracted data and critical appraisal judgments of one reviewer checked by a second reviewer. Other non-Cochrane reviews did also tend to favour two reviewers working independently (55%), but were also less likely to report exactly what was done (33% of reviews compared with 7% of HTA reviews and 2% of Cochrane reviews).

**Table 4:** Screening, extraction and risk of bias assessment in the included systematic reviews (the HTA data were collected for this study; all other data are from Page et al. [1], i.e. the data collected for 300 systematic reviews for a single month in 2004 and 2014)

Characteristic	2004 *n* = 300 [1]	HTA 2004 *n* = 23	HTA 2014 *n* = 30	Cochrane 2014 *n* = 45 [1]	2014 *n* = 300 [1]
Screening					
By at least two authors	NR	14 (61%)	22 (73%)	44 (98%)	200 (67%)
By one author, with a sample screened by a second	NR	2 (9%)	3 (10%)	1 (2%)	7 (2%)
By one author only	NR	2 (9%)	1 (3%)	0 (0%)	6 (2%)
Not reported / Unclear	NR	5 (22%)	4 (13%)	0 (0%)	87 (29%)
Empty review (no included / eligible studies)	NR	0/23 (0%)	2/30 (7%)	3/45 (7%)	4/300 (1%)
Extraction	NR	*n* = 23	*n* = 28	*n* = 42	*n* = 296
By at least two authors	NR	4 (17%)	5 (18%)	41 (98%)	163 (55%)
By one author, with data verified by a second	NR	14 (61%)	19 (68%)	0 (0%)	29 (10%)
By one author only	NR	1 (4%)	2 (7%)	0 (0%)	7 (2%)
Not reported / Unclear	NR	4 (17%)	2 (7%)	1 (2%)	97 (33%)
Quality assessment	- (67%)	23 (100%)	28 (100%)	42 (100%)	206 (70%)
By at least two authors	NR	5 (22%)	10 (36%)	37 (88%)	121/206 (59%)
By one author, with data verified by a second	NR	11 (48%)	12 (43%)	0 (0%)	6/206 (3%)
By one author only	NR	1 (4%)	0 (0%)	0 (0%)	3/206 (1%)
Not reported	NR	6 (26%)	6 (21%)	5 (12%)	76/206 (37%)
Risk of bias / quality assessment tool					
Cochrane (or modification)	NR	2 (9%)	12 (43%)	37 (88%)	77 (37%)
Jadad	NR	4 (17%)	0 (0%)	0(0%)	33 (11%)
Newcastle-Ottowa	NR	0	0 (0%)	1 (2%)	17 (8%)
QUADAS, QUADAS-2	NR	1 (4%)	5 (18%)	0 (0%)	8 (4%)
Own	NR	2 (9%)	3 (11%)	2 (5%)	38 (18%)
Other	NR	14 (61%)^a^	9 (32%)^a^	3 (7%)	53 (26%)
Not reported	NR	1 (4%)	1 (4%)	0 (0%)	6 (3%)
Selective reporting^b^	NR	0	10/28 (36%)	36/42 (86%)	70 (24%)

*HTA* Health Technology Assessment ^a^Especially CRD criteria, which are arguably as appropriate to UK HTA as the Cochrane risk of bias tool is to Cochrane reviews. ^b^Not always applicable, e.g. diagnostics: it is not a field in the QUADAS tool; *NR* Not reported

Inevitably, given the almost exclusive inclusion of RCTs or similar, the vast majority of Cochrane reviews used the Cochrane Risk of Bias tool to appraise included studies, while the non-Cochrane reviews, which included a much more diverse evidence base, used a broader range of tools. It is noteworthy, however, that HTA reviews had ceased to use the Jadad tool by 2014 (though other non-Cochrane reviews did continue to use this), and that a third of HTA reviews used critical appraisal criteria outlined by the York CRD guidance on conducting systematic reviews [15], which essentially had formed the basis of the UK HTA systematic review programme for many years. This was the HTA equivalent of Cochrane using the Cochrane risk of bias tool.

### Included studies, outcomes, analyses and findings

There were clear differences in the proportions of HTA reviews reporting full details of included and excluded studies between 2004 and 2014, and these reviews were superior to Cochrane and other non-Cochrane reviews in the reporting of these details (see Table 5). A substantially higher proportion of HTA reviews from 2014 also included grey literature (30%) than either Cochrane (18%) or non-Cochrane reviews (9%): this is likely due in part to the provision of unpublished data by companies or sponsors whose technologies were being assessed within the HTA reviews, but also because, as indicated in Table 3 above, more HTA reviews (50% compared with 20% of Cochrane reviews and 7% of non-Cochrane reviews) made efforts to search for this type of data.

**Table 5:** Review flow reporting, included studies, outcomes and analyses in the included systematic reviews (the HTA data were collected for this study; all other data are from Page et al. [1], i.e. the data collected for 300 systematic reviews for a single month in 2004 and 2014)

Characteristic	2004 *n* = 300 [1]	HTA 2004 *n* = 23	HTA 2014 *n* = 30	Cochrane 2014 *n* = 45 [1]	2014 *n* = 300 [1]
Review flow reporting					
PRISMA-like flow diagram (and text)	- (7%)	13 (57%)	29 (97%)	23 (51%)	206 (69%)
Text/tables or flowchart only	- (35%)	8 (35%)	1 (3%)	5 (11%)	20 (7%)
Partially reported	- (33%)	0	0 (0%)	7 (16%)	38 (13%)
Not reported	- (31%)	2 (9%)	0 (0%)	10 (22%)	36 (12%)
Reasons for exclusion of full text articles reported					
PRISMA-like flow diagram and text/tables	- (48%)	15 (65%)	27 (91%)	41 (91%)	211 (70%)
Partial (only reasons for some exclusions provided)	- (40%)	5 (22%)	1 (3%)	4 (9%)	28 (9%)
Not reported	- (17%)	3 (13%)	2 (7%)	0 (0%)	61 (20%)
Grey literature included in review					
Yes	NR	18 (78%)	9 (30%)*	8 (18%)	26 (9%)
Total number of included participants reported					
In main text	NR	2/23 (9%)	4/28 (14%)	39/42 (93%)	194/296 (66%)
In abstract	NR	1/23 (4%)	4/28 (14%)	37/42 (88%)	147/296 (50%)
At least one outcome reported in Methods	NR	21/23 (96%)	30 (100%)	45 (100%)	234 (78%)
A specified primary outcome	- (51%)	6 (26%)	16 (53%)	43(96%)	136/288 (47%)
Meta-analysis					
No (includes empty reviews or reviews with a statement that studies could not be combined)	NR	12 (52%)	15 (50%)	13 (29%)	111 (37%)
Yes	NR	11 (47%)	15 (50%)	32 (71%)	189 (63%)
Statistical heterogeneity investigated					
Quantitative	- (91%)	9/11 (82%)	13 (87%)	32/32 (100%)	175/189 (93%)
Not reported	NR	2/11 (18%)	1/15 (7%)	0 (0%)	12 (7%)
Risk of bias incorporated in meta-analysis	NR	0/23 (0%)	1/15 (7%)	4/32 (13%)	31/189 (16%)
Publication bias					
Formally assessed (Funnel plot etc.)	NR	0/23 (0%)	6/30 (20%)	7 (16%)	93 (31%)
Not assessed, but planned if sufficient studies	NR	1/23 (4%)	2/30 (7%)	28 (62%)	37 (12%)
Possibility discussed/considered in results, discussion etc.	- (31%)	0/23 (0%)	6/30 (20%)	29 (64%)	141 (47%)
GRADE assessment reported	NR	0 (0%)	0 (0%)	27 (60%)	32 (11%)
Limitations reported in Discussion/Conclusion					
At study level and review level	NR	11 (49%)	19 (63%)	32 (71%)	173 (58%)
Study-level only	NR	10 (44%)	6 (20%)	10 (22%)	67 (22%)
Review-level only	NR	0	3 (10%)	0 (0%)	27 (9%)
Not reported	NR	2 (7%)	2 (7%)		
Risk of bias / quality assessment reported					
In Abstract (Therapeutic reviews only)	NR	7/16 (44%) [63%‡]	8/14^a^ (57%) [79%^b^]	42 (93%)	99/164 (60%)

*HTA* Health Technology Assessment, *PRISMA* Preferred Reporting Items for Systematic Reviews and Meta-Analyses, *GRADE* Grading of Recommendations Assessment, Development and Evaluation (short GRADE); ^a^Excluding two empty reviews ^b^Percentage that include this in Executive or Scientific Summary

Only a very small proportion of HTA reviews reported the total number of participants from the included studies, contrasting sharply with the reporting of this characteristic in Cochrane and other non-Cochrane reviews. This is due in large part to a different convention applying in Health Technology Assessment reviews, i.e. the reporting of the range of numbers of participants from the included studies, e.g. ‘the five trials included between 50 and 150 patients’. Cochrane reviews were more likely to specify a primary outcome than Health Technology Assessment or other non-Cochrane reviews, and also more likely to perform a meta-analysis, although this is no surprise given the more restrictive parameters of Cochrane reviews, which are more likely to produce potentially homogenous data sets amenable to such analysis. This sample of HTA reviews was particularly poor in comparison with both Cochrane and other non-Cochrane reviews in addressing or even acknowledging publication bias. The 2014 Health Technology Assessment reviews also did not apply the Grading of Recommendations Assessment, Development and Evaluation (GRADE) process at all [16] and were less likely than Cochrane to mention the findings of the quality assessment process in the abstract. This latter issue might be due to two reasons: first, the brief abstracts for the HTA reviews do not include only the findings from the systematic review of the clinical evidence, but must also include the findings of the cost-effectiveness review and analyses that often accompany them. Second, Health Technology Assessment reviews also include a so-called ‘Scientific summary’ (2014) or ‘Executive summary’ (2004), a type of longer, structured abstract, in addition to the brief abstract. In the 2014 sample, only 8/14 (57%) of the therapeutic reviews with included studies mentioned risk of bias in the abstract but 11/14 (79%) included comments on quality assessment of the included studies in the Scientific summary.

## Discussion

The principal findings of this assessment are twofold. First, and previously undocumented, the standard of reporting is often comparable between UK Health Technology Assessment systematic reviews and Cochrane reviews, and the reporting in both appears to be more complete than other non-Cochrane reviews. Second, there were clear improvements in the conduct and reporting of Health Technology Assessment systematic reviews between 2004 and 2014, consistent with the findings of Page et al. regarding other ‘non-Cochrane’ and Cochrane systematic reviews [1].

Based on these data, the reporting of the Health Technology Assessment journal systematic reviews was comparable to Cochrane reviews and ‘superior’ to other non-Cochrane, across many characteristics: identification as a systematic review; registering of reviews and availability of protocols; conflicts of interest; reporting of the type of literature included; details of the database search strategies; searching of trial registries and grey literature; the use of PRISMA flow diagrams; the provision of details of any excluded studies; and the reporting of limitations at the level of the review and of the included studies. All of these elements have particular implications for informing decision-making, especially the importance of sourcing unpublished data [17, 18] and clarifying uncertainties within the evidence-base and review itself. This was all achieved despite the much broader scope of the Health Technology Assessment reviews compared with Cochrane reviews: Health Technology Assessment reviews covered a range of topics and included a diversity of study designs, while the Cochrane reviews exclusively covered therapeutic topics, mostly evaluating pharmacological interventions based almost exclusively on RCT or quasi-RCT evidence (100% of reviews compared with 53% of HTA reviews). Some of the other differences between Health Technology Assessment and Cochrane reviews, such as one reviewer in the former extracting and appraising studies, and a second checking those data and judgements, compared with double-extraction in Cochrane reviews, might also be explained by the relative scale of the task being faced by Health Technology Assessment reviewers compared with Cochrane reviewers. For example, the 2014 Health Technology Assessment reviews included reports with up to 200 studies, compared with 17 at the highest end of the interquartile range (IQR) in Cochrane reviews. Given the much larger number of included studies, the diverse questions and study designs, and the time constraints that govern HTA reviews (UK HTA reviews typically must be completed six to nine months after protocol approval) [19], unlike Cochrane reviews, some best practice ‘short-cut’ approaches are understandable. As a result, conduct and reporting is arguably much more straightforward within the narrower bounds of Cochrane systematic reviews, than in the broader, more diverse context in which certain non-Cochrane reviews are produced, especially Health Technology Assessment reviews.

At first glance, Health Technology Assessment reviews have weaker reporting than Cochrane reviews across several particular characteristics: total number of participants across the included studies; details of risk of bias assessments being reported in abstracts; specification of a primary outcome; a consideration of economics; the application of GRADE to ‘weigh’ evidence for the purposes of recommendations [16]; and publication bias. Some of these ‘deficiencies’ are easily explained and arguably should not be considered an issue. For example, Health Technology Assessment systematic reviews tend to report the range of participants across the included studies rather than a total, conduct economic evaluations as a distinct but related piece of work, and include risk of bias assessments in the larger Executive Summary rather than the brief Abstract, where the results of the accompanying cost-effectiveness analysis must also be reported. Health Technology Assessment systematic reviews are also not expected to develop recommendations based on the quality of the evidence – it is simply not a function of this type of systematic review– the responsibility for making recommendations lies with other groups in the HTA process [7, 19]. As a result, not one of the Health Technology Assessment reviews in 2014 reported using GRADE [16]. The one definite issue of poor conduct and reporting, however, concerns publication bias. This is simply almost never taken into account in Health Technology Assessment systematic reviews, but should be, without question.

The conduct and reporting of UK Health Technology Assessment journal reviews is therefore of the same standard as Cochrane reviews and generally more complete, and better, than many other reviews. Consequently, they should arguably not be ‘lumped together’ with other ‘non-Cochrane’ reviews, but considered as the equivalent of that perceived Cochrane ‘gold standard’. This raises the question why this set of reviews is so well-conducted and reported. The reason lies in the context in which they are produced: UK Health Technology Assessment reviews are commissioned by bodies associated with the UK Department of Health (National Institute of Health and Care Excellence [NICE] and the National Institute of Health Research [NIHR]) and with a specific policy-making and decision-making purpose and audience in mind [7]. While all reviews theoretically exist to support evidence-based practice [20, 21], not all reviews are produced within a process that exists to do so [19] and such a relationship is needed if research is to stand a chance of being influential [22]. So, while many reviews might be produced with the potential to influence practice, few are actually commissioned as an integral part of a process which is able to develop policy and inform practice [19]. Rather, as Page et al. have pointed out, it is to be suspected that many reviews are undertaken, and published, with rather less lofty aims in mind, such as a need to publish in order to secure promotions or funding for the authors [1]. There is also a concern that these authors do not have the skills to conduct and report systematic reviews as well as they should [1]. By contrast, the UK Health Technology Assessment journal reviews covered here often form part of a wider policy-making process – including taking into account cost-effectiveness of health technologies– with the aim of answering specific, policy-led questions, often about clinical decision-making and resource allocation, which can directly influence guidance and practice [7, 23]. The fact that Health Technology Assessment reviews are produced within such a context of evidence-based medicine, with the genuine potential to influence practice and be useful, means that the processes have to be rigorous, transparent and of the highest quality: ‘bias’ in the review needs to be minimized; the possibility of ‘misleading conclusions’, attributed by Page et al. to a sizeable number of non-Cochrane reviews, cannot be permitted in Health Technology Assessment reviews because there is too much riding on their findings [21, 23]. Given these implications, the HTA process has long included multiple specialist staff, as well as stakeholders outside of the review team who interrogate the work, and a rigorous editorial process for the Health Technology Assessment journal (which includes the requirement of a completed and checked PRISMA checklist). Such collaborations between funders, journals and academic institutions are all recommended by Page et al. as strategies to improve the conduct and reporting of systematic reviews generally [1]. This is an important consideration given that more than 8000 systematic reviews are published each year and, based on the sample of ‘non-Cochrane’ reviews analyzed by Page et al., a sizeable proportion are likely to be of ‘questionable quality’ [1].

Finally, there were apparent differences in conduct and reporting of systematic reviews published by the UK HTA programme, like all other reviews, between 2004 and 2014 across most domains, with the exception of the conduct and description of the searches for the reviews, which were of a very high standard in 2004 also. Overall, however, these differences are most likely the result of the publication and general acceptance of the PRISMA statement (Preferred Reporting Items for Systematic Reviews and Meta-analyses) as the relevant standard for the reporting of systematic reviews [3]. The 2009 PRISMA statement has doubtless led to improved reporting across all domains, but in this sample it unquestionably led to the following: higher numbers of Health Technology Assessment teams registering their review (70% of HTA reviews in 2014 compared with 0% in 2004) and making protocols available (90% compared with 17%); more complete reporting of limitations affecting the review itself, rather than the reporting of the limitations of included studies only (73% of HTA reviews in 2014 compared with 49% in 2004); and more complete reporting of decision-making regarding the inclusion (97% of HTA reviews in 2014 compared with 57% in 2004) and exclusion (91 and 65% respectively) of studies. Before 2009 the QUORUM (Quality of Reporting of Meta-analyses) statement existed to outline how inclusion and exclusion should be reported, but this was not applied in Health Technology Assessment journal reviews nearly as rigorously as the PRISMA statement and its related checklist [24]. It is also clear that conduct and reporting might have benefited from a greater standardization both of guidelines for systematic reviews and of the tools for the critical appraisal of included studies. Higher proportions of Health Technology Assessment reviews in 2014 were likely to report having followed guidelines in the conduct and reporting of the review than those in 2004 (63% did so compared with 24% in 2004) and, given the context in which they were produced, were as likely to cite the University of York Centre for Reviews and Dissemination (CRD) guidance [15] as the PRISMA statement [3]. The variety of available critical appraisal tools used in 2004 had been replaced by a smaller, more standardized set of tools by 2014, i.e. usage of the Cochrane Risk of Bias tool [25] increased from 9% in 2004 to 43% in 2014, and the use of ‘other’ tools ‘decreased’ from 61% in 2004 to 32% in 2014 (the vast majority of these ‘other’ tools in 2014 consisted of the application of CRD criteria for RCTs [15], which is ‘standard’ for Health Technology Assessment reviews). By 2014, a version of the QUADAS tool was being used for all of the HTA diagnostic reviews and the Jadad score had been dropped completely (from 17% of HTA reviews in 2004 to 0% in 2014), despite continuing to be used in 11% of other ‘non-Cochrane’ reviews.

The limitations of this work need to be acknowledged: this study only looked at a sample of HTA reviews from two years, in order to offer a direct comparison with the data presented by Page et al. [1], so results might be a little different if other years were selected. This work also only considered those characteristics previously examined by Page et al. [1]; different characteristics might have generated some different findings. Also, the data in Page et al. [1] were not re-checked, but taken as reported. Finally, some data are not straightforward (e.g. a search for ‘unpublished’ data might not be noted explicitly in the Methods of a Health Technology Assessment systematic review, but trial registers and grey literature are searched and often included and have much higher percentages), and so there might be some inconsistency in data interpretation across this study and the study by Page et al. [1] It should also be noted that this paper is not making generalizations about systematic reviews as a whole or all HTA systematic reviews.

## Conclusion

The aim of this research was to compare the reporting of systematic reviews published in the UK Health Technology Assessment journal series with the reporting of systematic reviews generally, and Cochrane reviews specifically. It found that UK Health Technology Assessment systematic reviews present standards of conduct and reporting equivalent to so-called ‘gold standard’ Cochrane reviews and superior to systematic reviews more generally. It therefore makes sense to view any systematic review produced within a genuine policy-context, and not just those from the UK Health Technology Assessment journal, as being on a par with Cochrane reviews and representing something quite different from other ‘non-Cochrane’ reviews. Indeed, knowing the purpose behind the production of a review – who commissioned the work, who funded the work, why the work was being undertaken – might be an obvious means by which users of reviews can ‘rate’ a systematic review’s likely reliability and rigour, [21] alongside assessments using the AMSTAR (Assessing the Methodological Quality of Systematic Reviews) or ROBIS tools [26, 27]. This is because reviews produced within a genuine policy-making context will have enjoyed resources and been through processes, given the implications of getting things wrong, that apply to few other reviews. This is not to say that other ‘non-Cochrane’ reviews produced outside of this context cannot adhere to high standards of conduct and reporting, of course they can; only that, based on the evidence as presented here, in the hierarchy of systematic reviews, UK Health Technology Assessment reviews deserve to be categorized as ‘gold standard’ alongside the more universally known Cochrane library of reviews. This is because the purpose behind these reviews resembles most closely the purpose originally intended for systematic reviews [20, 28]: to inform evidence-based medicine. Further, as pieces of research, given the context in which they are produced, they are more likely to be useful than many other reviews, in the sense that their findings almost certainly will be used, and all such research should be capable of being used [29, 30]. Again, they satisfy many of the requirements for genuinely useful research because they form part of an explicit policy-driven process in which time and money is expended with the specific aim of telling practitioners what to do and what not do.

## Additional file

Additional file 1: References of the included Health Technology Assessment monographs from 2004 (*n* = 22) and 2014 (*n* = 30). (DOCX 23 kb)

## Abbreviations

AMSTAR: Assessing the methodological quality of systematic reviews
CRD: Centre for reviews and dissemination
GRADE: Grading of recommendations assessment, development and evaluation
HTA: Health technology assessment
NICE: National institute of health and care excellence
NIHR: National institute of health research
NR: Not reported
PRISMA: Preferred reporting items for systematic reviews and meta-analyses
QUORUM: Quality of reporting of meta-analyses
RCT: Randomized controlled trial
ROBIS: Risk of bias in systematic reviews
UK: United Kingdom
