# Supplementary material for: Nature and reporting characteristics of UK health technology assessment systematic reviews
Source: BMC Med Res Methodol. 2018 May 8;18:35. doi: 10.1186/s12874-018-0498-6 (PMC5941703; doi:10.1186/s12874-018-0498-6)
Supplement: Supplementary file 1 — References of the included Health Technology Assessment monographs from 2004 (n = 22) and 2014 (n = 30). (DOCX 23 kb) [file 12874_2018_498_MOESM1_ESM.docx]

Additional file 1

2004 references

1. Avenell A, Broom J, Brown TJ, et al. Systematic review of the long-term effects and economic consequences of treatments for obesity and implications for health improvement. Health Technology Assessment (Winchester, England). 2004; 8: iii-iv, 1-182.

<http://www.journalslibrary.nihr.ac.uk/__data/assets/pdf_file/0014/65003/FullReport-hta8210.pdf>

1. Bridle C, Palmer S, Bagnall AM, et al. A rapid and systematic review and economic evaluation of the clinical and cost-effectiveness of newer drugs for treatment of mania associated with bipolar affective disorder. Health Technology Assessment (Winchester, England). 2004; 8: iii-iv, 1-187.

<http://www.journalslibrary.nihr.ac.uk/__data/assets/pdf_file/0020/65117/FullReport-hta8190.pdf>

1. Clark W, Jobanputra P, Barton P, et al. The clinical and cost-effectiveness of anakinra for the treatment of rheumatoid arthritis in adults: a systematic review and economic analysis. Health Technology Assessment (Winchester, England). 2004; 8: iii-iv, ix-x, 1-105.

<http://www.journalslibrary.nihr.ac.uk/__data/assets/pdf_file/0014/65120/FullReport-hta8180.pdf>

1. Czoski-Murray C, Warren E, Chilcott J, et al. Clinical effectiveness and cost-effectiveness of pioglitazone and rosiglitazone in the treatment of type 2 diabetes: a systematic review and economic evaluation. Health Technology Assessment (Winchester, England). 2004; 8: iii, ix-x, 1-91.

<http://www.journalslibrary.nihr.ac.uk/__data/assets/pdf_file/0003/65109/FullReport-hta8130.pdf>

1. Dalziel K, Round A, Stein K, et al. Effectiveness and cost-effectiveness of imatinib for first-line treatment of chronic myeloid leukaemia in chronic phase: a systematic review and economic analysis. Health Technology Assessment (Winchester, England). 2004; 8: iii, 1-120.

<http://www.journalslibrary.nihr.ac.uk/__data/assets/pdf_file/0016/65122/FullReport-hta8280.pdf>

1. Dundar Y, Boland A, Strobl J, et al. Newer hypnotic drugs for the short-term management of insomnia: a systematic review and economic evaluation. Health Technology Assessment (Winchester, England). 2004; 8: iii-x, 1-125.

<http://www.journalslibrary.nihr.ac.uk/__data/assets/pdf_file/0018/65133/FullReport-hta8240.pdf>

1. Garside R, Stein K, Wyatt K, et al. The effectiveness and cost-effectiveness of microwave and thermal balloon endometrial ablation for heavy menstrual bleeding: a systematic review and economic modelling. Health Technology Assessment (Winchester, England). 2004; 8: iii, 1-155.

<http://www.journalslibrary.nihr.ac.uk/__data/assets/pdf_file/0004/65119/FullReport-hta8030.pdf>

1. Green C, Colquitt JL, Kirby J, et al. Clinical and cost-effectiveness of once-daily versus more frequent use of same potency topical corticosteroids for atopic eczema: a systematic review and economic evaluation. Health Technology Assessment (Winchester, England). 2004; 8: iii,iv, 1-120.

<http://www.journalslibrary.nihr.ac.uk/__data/assets/pdf_file/0005/65138/FullReport-hta8470.pdf>

1. Green JM, Hewison J, Bekker HL, et al. Psychosocial aspects of genetic screening of pregnant women and newborns: a systematic review. Health Technology Assessment (Winchester, England). 2004; 8: iii, ix-x, 1-109.

<http://www.journalslibrary.nihr.ac.uk/__data/assets/pdf_file/0005/64841/FullReport-hta8330.pdf>

1. Hill R, Bagust A, Bakhai A, et al. Coronary artery stents: a rapid systematic review and economic evaluation. Health Technology Assessment (Winchester, England). 2004; 8: iii-iv, 1-242.

<http://www.journalslibrary.nihr.ac.uk/__data/assets/pdf_file/0003/65118/FullReport-hta8350.pdf>

1. Jones L, Griffin S, Palmer S, et al. Clinical effectiveness and cost-effectiveness of clopidogrel and modified-release dipyridamole in the secondary prevention of occlusive vascular events: a systematic review and economic evaluation. Health Technology Assessment (Winchester, England). 2004; 8: iii-iv, 1-196.

<http://www.journalslibrary.nihr.ac.uk/__data/assets/pdf_file/0020/65135/FullReport-hta8380.pdf>

1. Jones L, Hawkins N, Westwood M, et al. Systematic review of the clinical effectiveness and cost-effectiveness of capecitabine (Xeloda) for locally advanced and/or metastatic breast cancer. Health Technology Assessment (Winchester, England). 2004; 8: iii, xiii-xvi, 1-143.

<http://www.journalslibrary.nihr.ac.uk/__data/assets/pdf_file/0013/65110/FullReport-hta8050.pdf>

1. Kaltenthaler E, Vergel YB, Chilcott J, et al. A systematic review and economic evaluation of magnetic resonance cholangiopancreatography compared with diagnostic endoscopic retrograde cholangiopancreatography. Health Technology Assessment (Winchester, England). 2004; 8: iii, 1-89.

<http://www.journalslibrary.nihr.ac.uk/__data/assets/pdf_file/0015/65130/FullReport-hta8100.pdf>

1. Karnon J, Peters J, Platt J, et al. Liquid-based cytology in cervical screening: an updated rapid and systematic review and economic analysis. Health Technology Assessment (Winchester, England). 2004; 8: iii, 1-78.

<http://www.journalslibrary.nihr.ac.uk/__data/assets/pdf_file/0003/65127/FullReport-hta8200.pdf>

1. Knight C, Hind D, Brewer N, et al. Rituximab (MabThera) for aggressive non-Hodgkin's lymphoma: systematic review and economic evaluation. Health Technology Assessment (Winchester, England). 2004; 8: iii, ix-xi, 1-82.

<http://www.journalslibrary.nihr.ac.uk/__data/assets/pdf_file/0017/65123/FullReport-hta8370.pdf>

1. Main C, Palmer S, Griffin S, et al. Clopidogrel used in combination with aspirin compared with aspirin alone in the treatment of non-ST-segment-elevation acute coronary syndromes: a systematic review and economic evaluation. Health Technology Assessment (Winchester, England). 2004; 8: iii-iv, xv-xvi, 1-141.

<http://www.journalslibrary.nihr.ac.uk/__data/assets/pdf_file/0003/65136/FullReport-hta8400.pdf>

1. Mant J, McManus RJ, Oakes RA, et al. Systematic review and modelling of the investigation of acute and chronic chest pain presenting in primary care. Health Technology Assessment (Winchester, England). 2004; 8: iii, 1-158.

<http://www.journalslibrary.nihr.ac.uk/hta/volume-8/issue-2>

1. Mowatt G, Vale L, Brazzelli M, et al. Systematic review of the effectiveness and cost-effectiveness, and economic evaluation, of myocardial perfusion scintigraphy for the diagnosis and management of angina and myocardial infarction. Health Technology Assessment (Winchester, England). 2004; 8: iii-iv, 1-207.

<http://www.journalslibrary.nihr.ac.uk/__data/assets/pdf_file/0018/65124/FullReport-hta8300.pdf>

1. Pandor A, Eastham J, Beverley C, et al. Clinical effectiveness and cost-effectiveness of neonatal screening for inborn errors of metabolism using tandem mass spectrometry: a systematic review. Health Technology Assessment (Winchester, England). 2004; 8: iii, 1-121.

<http://www.journalslibrary.nihr.ac.uk/__data/assets/pdf_file/0003/65091/FullReport-hta8120.pdf>

1. Ross JR, Saunders Y, Edmonds PM, et al. A systematic review of the role of bisphosphonates in metastatic disease. Health Technology Assessment (Winchester, England). 2004; 8: 1-176.

<http://www.journalslibrary.nihr.ac.uk/__data/assets/pdf_file/0008/64997/FullReport-hta8040.pdf>

1. Shepherd J, Brodin H, Cave C, et al. Pegylated interferon alpha-2a and -2b in combination with ribavirin in the treatment of chronic hepatitis C: a systematic review and economic evaluation. Health Technology Assessment (Winchester, England). 2004; 8: iii-iv, 1-125.

<http://www.journalslibrary.nihr.ac.uk/__data/assets/pdf_file/0020/65126/FullReport-hta8390.pdf>

1. Warren E, Weatherley-Jones E, Chilcott J, et al. Systematic review and economic evaluation of a long-acting insulin analogue, insulin glargine. Health Technology Assessment (Winchester, England). 2004; 8: iii, 1-57.

<http://www.journalslibrary.nihr.ac.uk/__data/assets/pdf_file/0014/65102/FullReport-hta8450.pdf>

**2014 references**

1. Armstrong N, Burgers L, Deshpande S, et al. The use of fenestrated and branched endovascular aneurysm repair for juxtarenal and thoracoabdominal aneurysms: a systematic review and cost-effectiveness analysis. Health Technology Assessment (Winchester, England). 2014; 18: 1-66.

<http://www.journalslibrary.nihr.ac.uk/__data/assets/pdf_file/0012/131700/FullReport-hta18700.pdf>

1. Barton S, Karner C, Salih F, et al. Clinical effectiveness of interventions for treatment-resistant anxiety in older people: a systematic review. Health Technology Assessment (Winchester, England). 2014; 18: 1-59, v-vi.

<http://www.journalslibrary.nihr.ac.uk/__data/assets/pdf_file/0020/123932/FullReport-hta18500.pdf>

1. Bee P, Bower P, Byford S, et al. The clinical effectiveness, cost-effectiveness and acceptability of community-based interventions aimed at improving or maintaining quality of life in children of parents with serious mental illness: a systematic review. Health Technology Assessment (Winchester, England). 2014; 18: 1-250.

<http://www.journalslibrary.nihr.ac.uk/__data/assets/pdf_file/0019/106453/FullReport-hta18080.pdf>

1. Brazier J, Connell J, Papaioannou D, et al. A systematic review, psychometric analysis and qualitative assessment of generic preference-based measures of health in mental health populations and the estimation of mapping functions from widely used specific measures. Health Technology Assessment (Winchester, England). 2014; 18: vii-viii, xiii-xxv, 1-188.

<http://www.journalslibrary.nihr.ac.uk/__data/assets/pdf_file/0006/118176/FullReport-hta18340.pdf>

1. Brazzelli M, Cruickshank M, Kilonzo M, et al. Clinical effectiveness and cost-effectiveness of cholecystectomy compared with observation/conservative management for preventing recurrent symptoms and complications in adults presenting with uncomplicated symptomatic gallstones or cholecystitis: a systematic review and economic evaluation. Health Technology Assessment (Winchester, England). 2014; 18: 1-101, v-vi.

<http://www.journalslibrary.nihr.ac.uk/__data/assets/pdf_file/0009/124749/FullReport-hta18550.pdf>

1. Bryant M, Ashton L, Brown J, et al. Systematic review to identify and appraise outcome measures used to evaluate childhood obesity treatment interventions (CoOR): evidence of purpose, application, validity, reliability and sensitivity. Health Technology Assessment (Winchester, England). 2014; 18: 1-380.

<http://www.journalslibrary.nihr.ac.uk/__data/assets/pdf_file/0005/124196/FullReport-hta18510.pdf>

1. Burch J, Rice S, Yang H, et al. Systematic review of the use of bone turnover markers for monitoring the response to osteoporosis treatment: the secondary prevention of fractures, and primary prevention of fractures in high-risk groups. Health Technology Assessment (Winchester, England). 2014; 18: 1-180.

<http://www.journalslibrary.nihr.ac.uk/__data/assets/pdf_file/0013/109201/FullReport-hta18110.pdf>

1. Campbell F, Thokala P, Uttley LC, et al. Systematic review and modelling of the cost-effectiveness of cardiac magnetic resonance imaging compared with current existing testing pathways in ischaemic cardiomyopathy. Health Technology Assessment (Winchester, England). 2014; 18: 1-120.

<http://www.journalslibrary.nihr.ac.uk/__data/assets/pdf_file/0014/126311/FullReport-hta18590.pdf>

1. Colquitt JL, Mendes D, Clegg AJ, et al. Implantable cardioverter defibrillators for the treatment of arrhythmias and cardiac resynchronisation therapy for the treatment of heart failure: systematic review and economic evaluation. Health Technology Assessment (Winchester, England). 2014; 18: 1-560.

<http://www.journalslibrary.nihr.ac.uk/__data/assets/pdf_file/0005/124745/FullReport-hta18560.pdf>

1. Farmer AJ, Stevens R, Hirst J, et al. Optimal strategies for identifying kidney disease in diabetes: properties of screening tests, progression of renal dysfunction and impact of treatment - systematic review and modelling of progression and cost-effectiveness. Health Technology Assessment (Winchester, England). 2014; 18: 1-128.

<http://www.journalslibrary.nihr.ac.uk/__data/assets/pdf_file/0008/112796/FullReport-hta18140.pdf>

1. Fleeman N, Pilkington G, Dundar Y, et al. Allopurinol for the treatment of chronic kidney disease: a systematic review. Health Technology Assessment (Winchester, England). 2014; 18: 1-77, v-vi.

<http://www.journalslibrary.nihr.ac.uk/__data/assets/pdf_file/0006/120777/FullReport-hta18400.pdf>

1. Frampton G, Harris P, Cooper K, et al. The clinical effectiveness and cost-effectiveness of second-eye cataract surgery: a systematic review and economic evaluation. Health Technology Assessment (Winchester, England). 2014; 18: 1-205, v-vi.

<http://www.journalslibrary.nihr.ac.uk/__data/assets/pdf_file/0013/130144/FullReport-hta18680.pdf>

1. Frampton GK, Harris P, Cooper K, et al. Educational interventions for preventing vascular catheter bloodstream infections in critical care: evidence map, systematic review and economic evaluation. Health Technology Assessment (Winchester, England). 2014; 18: 1-365.

<http://www.journalslibrary.nihr.ac.uk/__data/assets/pdf_file/0012/113034/FullReport-hta18150.pdf>

1. Hartwell D, Cooper K, Frampton GK, et al. The clinical effectiveness and cost-effectiveness of peginterferon alfa and ribavirin for the treatment of chronic hepatitis C in children and young people: a systematic review and economic evaluation. Health Technology Assessment (Winchester, England). 2014; 18: i-xxii, 1-202.

<http://www.journalslibrary.nihr.ac.uk/__data/assets/pdf_file/0011/128999/FullReport-hta18650.pdf>

1. Harvey NC, Holroyd C, Ntani G, et al. Vitamin D supplementation in pregnancy: a systematic review. Health Technology Assessment (Winchester, England). 2014; 18: 1-190.

<http://www.journalslibrary.nihr.ac.uk/__data/assets/pdf_file/0004/121765/FullReport-hta18450.pdf>

1. Holmes M, Rathbone J, Littlewood C, et al. Routine echocardiography in the management of stroke and transient ischaemic attack: a systematic review and economic evaluation. Health Technology Assessment (Winchester, England). 2014; 18: 1-176.

<http://www.journalslibrary.nihr.ac.uk/__data/assets/pdf_file/0017/113246/FullReport-hta18160.pdf>

1. Kaltenthaler E, Pandor A, Wong R. The effectiveness of sexual health interventions for people with severe mental illness: a systematic review. Health Technology Assessment (Winchester, England). 2014; 18: 1-74.

<http://www.journalslibrary.nihr.ac.uk/__data/assets/pdf_file/0010/97498/FullReport-hta18010.pdf>

1. Leaviss J, Sullivan W, Ren S, et al. What is the clinical effectiveness and cost-effectiveness of cytisine compared with varenicline for smoking cessation? A systematic review and economic evaluation. Health Technology Assessment (Winchester, England). 2014; 18: 1-120.

<http://www.journalslibrary.nihr.ac.uk/__data/assets/pdf_file/0019/117811/FullReport-hta18330.pdf>

1. Livingston G, Kelly L, Lewis-Holmes E, et al. A systematic review of the clinical effectiveness and cost-effectiveness of sensory, psychological and behavioural interventions for managing agitation in older adults with dementia. Health Technology Assessment (Winchester, England). 2014; 18: 1-226, v-vi.

<http://www.journalslibrary.nihr.ac.uk/__data/assets/pdf_file/0014/120551/FullReport-hta18390.pdf>

1. Llewellyn A, Norman G, Harden M, et al. Interventions for adult Eustachian tube dysfunction: a systematic review. Health Technology Assessment (Winchester, England). 2014; 18: 1-180, v-vi.

<http://www.journalslibrary.nihr.ac.uk/__data/assets/pdf_file/0004/122746/FullReport-hta18460.pdf>

1. Longworth L, Yang Y, Young T, et al. Use of generic and condition-specific measures of health-related quality of life in NICE decision-making: a systematic review, statistical modelling and survey. Health Technology Assessment (Winchester, England). 2014; 18: 1-224.

<http://www.journalslibrary.nihr.ac.uk/__data/assets/pdf_file/0008/108368/FullReport-hta18090.pdf>

1. Loveman E, Jones J, Clegg AJ, et al. The clinical effectiveness and cost-effectiveness of ablative therapies in the management of liver metastases: systematic review and economic evaluation. Health Technology Assessment (Winchester, England). 2014; 18: vii-viii, 1-283.

<http://www.journalslibrary.nihr.ac.uk/__data/assets/pdf_file/0010/105004/FullReport-hta18070.pdf>

1. Mowatt G, Hernandez R, Castillo M, et al. Optical coherence tomography for the diagnosis, monitoring and guiding of treatment for neovascular age-related macular degeneration: a systematic review and economic evaluation. Health Technology Assessment (Winchester, England). 2014; 18: 1-254.

<http://www.journalslibrary.nihr.ac.uk/__data/assets/pdf_file/0005/131369/FullReport-hta18690.pdf>

1. Simpson EL, Kearns B, Stevenson MD, et al. Enhancements to angioplasty for peripheral arterial occlusive disease: systematic review, cost-effectiveness assessment and expected value of information analysis. Health Technology Assessment (Winchester, England). 2014; 18: 1-252.

<http://www.journalslibrary.nihr.ac.uk/__data/assets/pdf_file/0003/108534/FullReport-hta18100.pdf>

1. Snowsill T, Huxley N, Hoyle M, et al. A systematic review and economic evaluation of diagnostic strategies for Lynch syndrome. Health Technology Assessment (Winchester, England). 2014; 18: 1-406.

<http://www.journalslibrary.nihr.ac.uk/__data/assets/pdf_file/0005/125978/FullReport-hta18580.pdf>

1. Stevenson M, Gomersall T, Lloyd Jones M, et al. Percutaneous vertebroplasty and percutaneous balloon kyphoplasty for the treatment of osteoporotic vertebral fractures: a systematic review and cost-effectiveness analysis. Health Technology Assessment (Winchester, England). 2014; 18: 1-290.

<http://www.journalslibrary.nihr.ac.uk/__data/assets/pdf_file/0017/114317/FullReport-hta18170.pdf>

1. Wardlaw J, Brazzelli M, Miranda H, et al. An assessment of the cost-effectiveness of magnetic resonance, including diffusion-weighted imaging, in patients with transient ischaemic attack and minor stroke: a systematic review, meta-analysis and economic evaluation. Health Technology Assessment (Winchester, England). 2014; 18: 1-368, v-vi.

<http://www.journalslibrary.nihr.ac.uk/__data/assets/pdf_file/0011/117002/FullReport-hta18270.pdf>

1. Westwood M, Joore M, Whiting P, et al. Epidermal growth factor receptor tyrosine kinase (EGFR-TK) mutation testing in adults with locally advanced or metastatic non-small cell lung cancer: a systematic review and cost-effectiveness analysis. Health Technology Assessment (Winchester, England). 2014; 18: 1-166.

<http://www.journalslibrary.nihr.ac.uk/__data/assets/pdf_file/0018/117621/FullReport-hta18320.pdf>

1. Westwood M, van Asselt T, Ramaekers B, et al. KRAS mutation testing of tumours in adults with metastatic colorectal cancer: a systematic review and cost-effectiveness analysis. Health Technology Assessment (Winchester, England). 2014; 18: 1-132.

<http://www.journalslibrary.nihr.ac.uk/__data/assets/pdf_file/0003/127659/FullReport-hta18620.pdf>

1. Whiting P, Al M, Burgers L, et al. Ivacaftor for the treatment of patients with cystic fibrosis and the G551D mutation: a systematic review and cost-effectiveness analysis. Health Technology Assessment (Winchester, England). 2014; 18: 1-106.

<http://www.journalslibrary.nihr.ac.uk/__data/assets/pdf_file/0004/114565/FullReport-hta18180.pdf>
